# Mammary Myofibroblastoma: Report of a Case and Review of the Literature

**DOI:** 10.7759/cureus.32371

**Published:** 2022-12-09

**Authors:** Nektarios Koufopoulos, Alina-Roxani Gouloumis, Dionysios T Dimas, Adamantia Kontogeorgi, Kyparissia Sitara, Ioannis Boutas

**Affiliations:** 1 2nd Department of Pathology, National and Kapodistrian University of Athens, “Attikon” University Hospital, Athens, GRC; 2 Breast Unit, Athens Medical Center, Psychiko Clinic, Athens, GRC; 3 3rd Department of Obstetrics and Gynecology, National and Kapodistrian University of Athens, “Attikon” University Hospital, Athens, GRC; 4 Department of Internal Medicine, “Elpis” General Hospital of Athens, Athens, GRC; 5 Breast Unit, Rea Maternity Hospital, Athens, GRC

**Keywords:** invasive lobular carcinoma, metaplastic carcinoma, differential diagnosis, breast, myofibroblastoma

## Abstract

Mammary myofibroblastoma is a benign mesenchymal tumor composed of fibroblasts, myofibroblasts, and a variable number of adipocytes. Mammary myofibroblastoma usually occurs in men of older age and is less common in postmenopausal women. It may also happen in extramammary sites along the milk line. In this instance, it is referred to as mammary-type myofibroblastoma. Rarely multifocal and bilateral tumors have been described. Clinically and radiologically, it can be misinterpreted as a malignant tumor due to its rarity. Size usually does not exceed 3 cm. The diagnosis requires clinicopathological correlation with morphological and immunohistochemical evaluation, especially in limited biopsy specimens. We herewith describe a rare case of mammary myofibroblastoma in a 37-year-old female patient. We also review the literature focusing on the potential differential diagnostic issues and discuss this tumor's ultrastructural and cytogenetic findings.

## Introduction

Mammary myofibroblastoma (MFB) is a benign mesenchymal tumor composed of fibroblasts and myofibroblasts and a variable number of adipocytes that was first described in 1987 by Wargotz et al. [1]. MFB occurs in the breast and less commonly in extramammary sites, including soft tissue and the female genital tract, usually along the milk line [2]. In the latter case, it is referred to as mammary-type MFB. It occurs most commonly in men of older age and postmenopausal women showing a male patient predilection [3,4]. It has been reported in men with gynaecomastia [5], treated for prostate cancer [6], and in transgender patients on feminizing hormones [7]. In addition, some examples of this tumor have been reported in association with invasive breast carcinoma [6], after radiation therapy for ductal carcinoma in situ [8], and at the site of a surgical scar [3,9]. On rare occasions, it may be multiple and/or bilateral [10,11]. It can be misinterpreted as a clinically and radiologically malignant tumor [12]. Size usually does not exceed 3 cm, ranging from 2 mm to 18 cm [3,13,14]. The diagnosis may prove challenging, especially in needle core biopsy material. Only a few cases have been described in the English literature. In this manuscript, we present a case of mammary MFB in a premenopausal 37-year-old female patient, and we review the literature focusing on potential differential diagnostic issues. We also discuss the ultrastructural and cytogenetic findings of this rare entity. This article was previously presented as a meeting abstract at the 2018 XXXII Congress of the International Academy of Pathology on October 14-18, 2018.

## Case presentation

**Figure 1:**
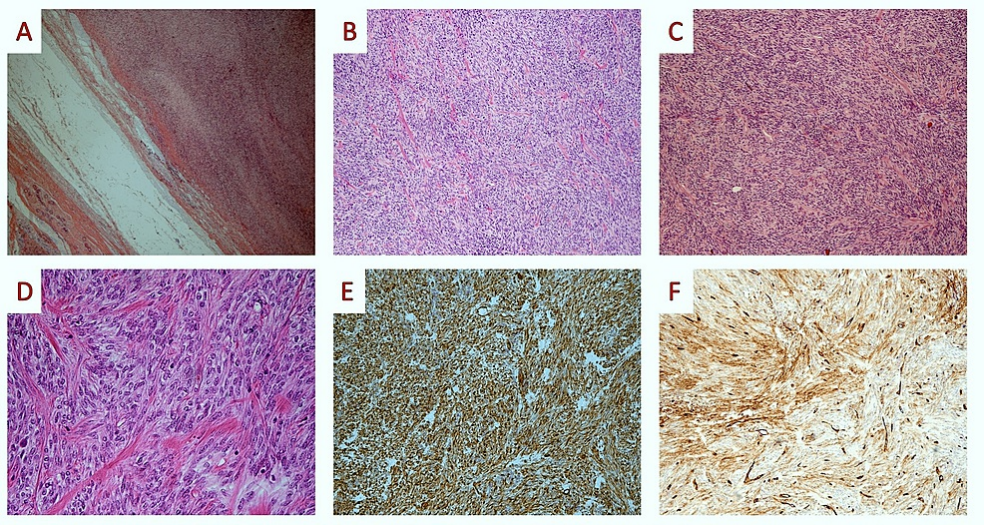
Histopathological images (A; H&E X 04): On microscopic examination, the tumor lacked a capsule but was well circumscribed from the surrounding breast parenchyma. (B, C; H&E X 10): On low-power examination, the tumor consisted of fascicles of uniform, bland short spindle cells. (D; H&E X 40): On higher power examination, tumor cells were short spindle-shaped, with a moderate amount of pale to eosinophilic cytoplasm, oval nuclei, and a low mitotic count. Some keloidal-like, brightly eosinophilic collagen fibers can be seen. (E, F; Vimentin, CD34 X 10): Tumor cells showed positive staining for Vimentin and CD34.

A 37-year-old patient was admitted to our hospital due to a painless, solitary, slowly growing, palpable mass in her right breast. The tumor was firm in consistency, non-tender, and freely movable on clinical examination. A breast ultrasound revealed an oval, circumscribed, homogeneously isoechoic mass with a maximum diameter of 3 cm. Mammography and breast magnetic resonance imaging (MRI) revealed an oval, circumscribed, hyperdense mass. Fine needle aspiration cytology was negative for malignancy, and biopsy revealed a spindle cell lesion without significant atypia or mitotic activity that was immunohistochemically positive for Vimentin and CD34 and negative for CKAE1/AE3, CK8/18, and P63. The descriptive diagnosis was “spindle cell lesion lacking atypia or mitoses possibly benign, but a low malignant potential lesion cannot be excluded." The tumor was excised with wide margins. On gross examination, the tumor was well-circumscribed, solid, and grey-white, with a maximum diameter of 3.2 cm. On microscopic examination, the tumor consisted of fascicles of uniform, bland, short spindle cells with a moderate amount of pale to eosinophilic cytoplasm. The nuclei were oval. Mitotic figures were less than 2 per 10 high-power fields (HPF). Numerous bands of keloidal-like eosinophilic collagen separating tumor cells were present. There were few entrapped mammary glands at the tumor periphery. The immunohistochemical study was positive for Vimentin and CD-34 and negative for CKAE-1/AE-3, CK-8/18, S-100, P-63, SMA, Desmin, and Rb. Ki-67 stained around 2% of tumor nuclei (Figure 1).

Our findings were consistent with mammary MFB. The surgical margins were tumor free. The patient received no further treatment and is alive with no evidence of recurrence or metastasis 55 months after surgery.

## Discussion

MFB is a rare benign spindle cell tumor showing striking morphologic, immunohistochemical, and genetic similarities with spindle cell lipoma [4,15]. Clinically, MFB has a benign clinical behavior. Recurrence is unlikely if clear resection margins are achieved. Malignant transformation or metastasis has not been documented in the English literature. Imaging findings are non-specific and sometimes suggest fibroadenoma [3,15]. It appears as a circumscribed, hyperdense, or isodense mass on mammography. On ultrasound, it usually appears well-circumscribed and hypoechoic. Also, dynamic contrast-enhanced breast MRI has a circumscribed margin and isointense on T1-weighted images and hyperintense on fat-suppressed T2-weighted images [12].

On gross examination, it is a well-circumscribed, not encapsulated solid lump with a firm, whitish-gray nodular or whorled cut surface [2,3]. Sometimes, it may be multilobulated [16]. On microscopic examination, MFB is well-circumscribed without a true capsule. It is composed of short to elongated spindle cells arranged in short haphazard intersecting fascicles admixed with bands of hyalinized, brightly eosinophilic collagen and variable amounts of fat. Tumor cells are typically uniform with bland cytologic features. Only a minority of cases (around 10%) display cytologic atypia [2]. Mitoses are found rarely, and atypical mitoses and necrosis are absent. In some instances, mammary MFB may show smooth muscle and rarely cartilaginous or osseous differentiation [17,18]. Several variants have been described, including fibrous [19], cellular [9], infiltrating [20], myxoid [15], deciduoid [21], lipomatous [22], epithelioid [23], with hemangiopericytoma-like pattern [24], and atypical [25].

Immunohistochemically, tumor cells show positive staining for Vimentin, Desmin, ER, PR, AR, BCL2, CD10, CD99, and CD34 [2,26,27]. In examples with smooth muscle differentiation, H-caldesmon is expressed [28,29]. Cytokeratins, S100, p63 CD117, and Rb, lack immunohistochemical staining [2,3].

The differential diagnosis of mammary MFB includes invasive lobular carcinoma (ILC), metaplastic spindle cell breast carcinoma, desmoid-type fibromatosis, nodular fasciitis, pseudoangiomatous stromal hyperplasia, solitary fibrous tumor, and spindle cell lipoma. ILC, metaplastic spindle cell carcinoma (MSCC), fibromatosis like metaplastic carcinoma, metaplastic matrix-producing carcinoma (MMPC), desmoid fibromatosis, nodular fasciitis, pseudoangiomatous stromal hyperplasia, and solitary fibrous tumor enter the differential diagnosis since they may simulate histologically different variants of mammary MFB. Like ILC, the epithelioid variant of mammary MFB can display histologically a single file pattern and shows positive ER and PR staining in most cases. MSCC and fibromatosis-like metaplastic carcinoma may simulate the histological features of MFB. Also, in rare cases with cartilaginous or osseous components, MMPC may enter the differential diagnosis [30,31].

In most cases, MFB has a well-circumscribed border, with a few cases showing an infiltrative growth pattern. It is composed of bland cells with a minority of instances displaying cytologic atypia, which can sometimes be prominent, focal, or multifocal, although never diffuse [2]. Mitoses are few (less than 2 per 10 HPFs). The presence of an in situ component favors the diagnosis of carcinoma [15]. The fibromatosis-like metaplastic carcinoma can be almost impossible to distinguish from MFB based on morphology since it has bland cytological characteristics. In this context, immunohistochemical stains for keratins, p63, CD34, desmin, and Rb can assist in the diagnosis. Lack of staining for cytokeratins p63 and Rb1 combined with positive CD34 and desmin expression favors the diagnosis of mammary MFB. Desmoid-type fibromatosis consists of a proliferation of long, sweeping fascicles with infiltrative borders. It shows the nuclear expression of β-catenin and is CD34 negative. Nodular fasciitis consists of a loose storiform proliferation of bland spindle cells with occasional mitoses, scattered inflammatory cells, and extravasated erythrocytes can sometimes be present. In contrast to mammary MFB, nodular fasciitis is CD34 and desmin negative. Pseudoangiomatous stromal hyperplasia does not usually form a discrete mass and histologically shows empty, anastomosing CD34, ER, PR positive vascular-like spaces. In the rare case when MFB has a hemangiopericytoma-like pattern mimicking a solitary fibrous tumor, the diagnostic problem is complicated because both tumors express CD34. A stain for STAT6 (positive in the solitary fibrous tumor) will solve the diagnostic problem [32,33]. Spindle cell lipoma is characterized by a predominant fat component and lacks desmin expression. However, the differential diagnosis is not crucial since both entities are benign and share similar chromosomal abnormalities. Electron microscopy has revealed a variable admixture of fibroblasts, myofibroblasts, smooth muscle cells, and undifferentiated mesenchymal cells in the cases studied [3]. Cytogenetic studies have revealed a total loss of 13q14 region and partial loss of 16q. These alterations are similar to those described in spindle cell lipoma [4]. Regarding treatment, mammary MFB is a benign lesion that does not recur or metastasize. In this context, surgical excision with adequate margins is the treatment of choice [16].

## Conclusions

In summary, we presented a case of mammary MFB in a premenopausal woman, and we reviewed the English literature focusing on this rare entity's potential differential diagnostic issues. Pathologists should be aware of the broad morphologic spectrum exhibited by mammary MFB to avoid a misdiagnosis of malignancy. In difficult cases, using appropriate immunohistochemical stains can help in the precise diagnosis. We have also discussed the electron microscopy cytogenetic findings and treatment of this rare entity.
